# Normative data of bone Mineral Density in healthy population of Tehran, Iran: A Cross sectional study

**DOI:** 10.1186/1471-2474-6-38

**Published:** 2005-07-02

**Authors:** Bagher Larijani, Arash Hossein-Nezhad, Alireza Mojtahedi, Mohammad Pajouhi, Mohammad H Bastanhagh, Akbar Soltani, Seyed-Zahra Mirfezi, Roya Dashti

**Affiliations:** 1 Endocrinology & Metabolism Research Center, Tehran University of Medical Sciences, Fifth floor, Dr. Shariati Hospital, North Kargar Ave, Tehran, 14114, Iran

## Abstract

**Background:**

Osteoporosis is a major problem and is a hidden epidemic disease in the world. Early diagnosis by measurement of Bone Mineral Density (BMD) and treatment can prevent and reduce disease complications, especially fractures. As there is no comprehensive study in Iran, this study designed to assess BMD discrepancy in 20–69 yr Tehran population as well as prevalence of osteoporosis and osteopenia.

**Methods:**

553 people (34% men, 66%women) from 50 Blocks in Tehran randomly selected. The assessment of BMD in spine and femur region performed through DXA method. All subjects clinically examined and their BMIs determined.

**Results:**

The average spinal BMD score in men were more than in women. The peak bone mass of spine bone both in men and women occurred during 20–29 yr and reduction began from the age of 40. At the age of 60 to 69, loose of bone density was 19.6% in lumbar spine and 18.5% in femur of women and also 7.9% in lumbar spine and 14.6% in femur of men. Prevalence of osteoporosis in this age group in lumbar spine and femur was 32.4% and 5.9% in women and 9.4% and 3.1% in men respectively.

**Conclusion:**

In all age groups, peak bone mass was lower than European or American population, whereas the rate of bone loss was as much as the some population and actually this process justifies the prevalence of osteoporosis and osteopenia in Tehran population.

## Background

Osteoporosis is the most common metabolic disease of bone which is known by deficit in bone mineral density and skeletal micro destruction that increases risk of bone fracture [1-3]. The importance of BMD is, in diagnosis of osteoporosis and prevention of bone fractures and its consequent disability [1,2]. BMD depends on age, disease, genetic, mechanical factors, nutrition, and the body hormones effects[4] Studies showed that the prevalence of osteoporosis the age of over 70, it increases to 87% [5,6]. In Thailand's over 70 yr women, prevalence of disease was 50% [6]. In the UK, 1/3 of women and 1/12 of men affected from osteoporosis [7]. The investigations showed that in the next 50 years, considering the population growth in the old people in Asia, South America and Africa, it is expected almost 75% of these fractures occur in progressing countries [1]. A study, that recently performed in Iran showed, osteoprotic fracture data was important public health problem in Iran [8].

For reliable interpretation of individual BMD data, however, they need to be expressed in relation to established normative data. Comparisons can be made either in terms of the age-matched standard deviation score, use of the *T *score, which indicates deviation from the mean BMD of a young normal population [9]. For this reason, comparison of *T *scores yields the best available information on the extent of osteoporotic bone loss and the associated fracture risk.

In clinical practice, individual BMD values are compared with a reference value. For diagnostic purposes, a panel convened by the WHO proposed to define osteoporosis on the basis of the T-score, According to this categories, a *T *score between -1 and -2.5 is indicative of osteopenia, while a *T *score -'-2· 5 reflects osteoporosis [9-12]. Despite its limitations [13-15]; this definition is currently applied worldwide. However, the normal values provided by manufacturers may not be fully representative of specific local populations. In fact, BMD is influenced by several variables, including genetic and environmental factors [16,17]. Thus, reference ranges may vary in different populations [18-23], and [24].

Early diagnosis of osteoporosis by assessment of bone density can prevent its complications, especially fractures. Bone density relates to many items like race, genetic, sex, environmental factors and nutrition. In order to define osteoporosis and osteopenia, knowledge of the reference data of bone mineral density (BMD) is important. As there is not any reference data on BMD and osteopenia and osteoporosis in Iran, we decided to perform a first national comprehensive study which it's aims of this study were to determine normal values of bone mineral density at lumbar vertebras and neck of femur and determine prevalence of osteopenia and osteoporosis for Iranian normal population.

## Methods

This was a cross sectional study. In duration of 6 months, 553 subjects were selected among the men and women of 20_ 69 yr of Tehran. The individuals selected based on randomized clustered sampling from 50 blocks in Tehran. For selection of clusters, whole Tehrani's population on base of distribution of them, divided to many clusters. From all clusters 50 of them were randomly selected and after that, in each block, home's numbers whose numbers were twin were selected on based of exclusion and inclusion criteria, in each home 1 person were selected, until the individuals in each group reach to 24 persons. The exclusion criteria were selected from diseases and drugs that may have effects on metabolism of bone and Vitamin D. Smoking, alcohol, pregnancy, breast-feeding during the study, Professional sport, General conditions and Immobility also were on exclusion criteria.

Healthy individual were selected if they did not have any problem that affect on bone or Vitamin D metabolism based on exclusion criteria. Previous and current diseases, drugs, and habits were determined by personal interview and were evaluated by nurses through the interview. 750 individuals were invited for this study, 533 individuals came for assessment. After 2 times recall, there is not any significant difference between the mean age and sex distribution of individuals who came and who did not come. 217 subjects refused to participate in this study.

The study protocol was approved by research ethic committee of Endocrinology and Metabolism Research Centre (EMRC) and the data gathered by cluster random sampling. The subjects with osteoporosis were referred to the EMRC osteoporosis clinic in the Shariati Hospital for treatment.

Having received the letters of consent, the related questionners were completed and clinical examinations such as height and weight were carried out. BMD was measured by DXA using Lunar DPX-MD device (Lunar Corporation, Madison, Wisconsin, 53713. USA). The DXA device was calibrated daily and weekly by using appropriated phantoms methods. To assess BMD, second to fourth lumbar spine and from the femur bone (neck, trochanter and the whole femur), bone density was calculated based on gr/cm^2^.

SPSS (ver 11.5) was used for data analysis. To compare the mean, the student T test was used and for comparing frequency of variable between groups Chi-square was used.

## Results

553 subjects (34% men, 66% women) between 20 to 69 yr (mean ± SD, 44.07 ± 12.68) participated in the study. Basic characteristics of the subjects showed that in Table 1. There was no significant difference in the spinal BMD of women between the age groups 20–29 and 30–39 (Table 2).

**Table 1:** Basic characteristic of the subjects in each age and sex groups

**Age groups**		**Numbers**	**Weight**(Kg)	**Height**(cm)	**BMI**(Kg/m^2^)
			mean ± SD	mean ± SD	mean ± SD
**20–29**	Women	44	60.1 ± 11.4	160 ± 4.38	23.45 ± 4.1
	Men	27	71.27 ± 12.7	173.58 ± 6.16	23.04 ± 3.95
**30–39**	Women	104	67.43 ± 11.34	156.46 ± 8.29	27.72 ± 5.7
	Men	38	77.68 ± 15.99	171.51 ± 7.6	26.29 ± 4.45
**40–49**	Women	98	70.96 ± 17.58	157.2 ± 6.31	28.61 ± 6.01
	Men	48	76.37 ± 10.85	168.11 ± 6.11	27.02 ± 3.55
**50–59**	Women	82	68.27 ± 11.68	154.04 ± 6.19	28.73 ± 4.74
	Men	42	73.66 ± 12.99	166.07 ± 6.15	66.67 ± 4.33
**60–69**	Women	36	65.67 ± 10.09	152.97 ± 6.72	28.12 ± 4.45
	Men	34	72.94 ± 11.73	16312 ± 6.07	27.41 ± 4.23

**Table 2:** Mean BMDs at the Lumbar Spine and Femur for each Age and Sex groups

**Measurement Site**	**Mean BMD in femur (gr/cm^2^)**		**Mean BMD in Lumbar Spine(gr/cm**^2^**)**	
**Age group**	Women	Men	Women	Men
**20–29**	0.962 ± 0.132	1.098 ± 0.15	**1.198 ± 0.1132	1.209 ± 0.132
**30–39**	1.022 ± 0.122	1.042 ± 0.146	1.206 ± 0.1249	1.216 ± 0.1414
**40–49**	0.968 ± 0.120	1.009 ± 0.144	1.158 ± 0.148	1.202 ± 0.176
**50–59**	0.9179 ± 0.120	0.966 ± 0.206	1.024 ± 0.178	1.120 ± 0.129
**60–69**	0.833 ± 0.111	0.935 ± 0.105	0.982 ± 0.161	1.117 ± 0.155

** Mean ± SD

**Figure 1:**
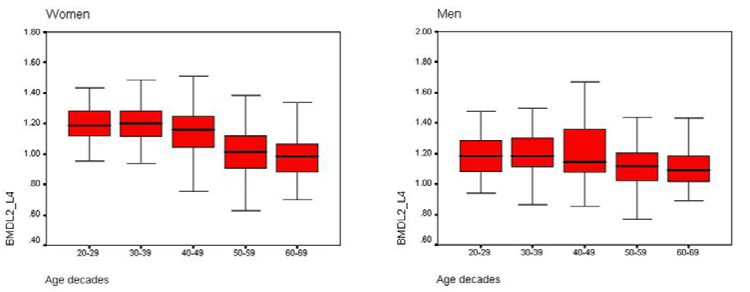
BMD of lumbar spine within age decades and sex (g/cm^2^).

**Figure 2:**
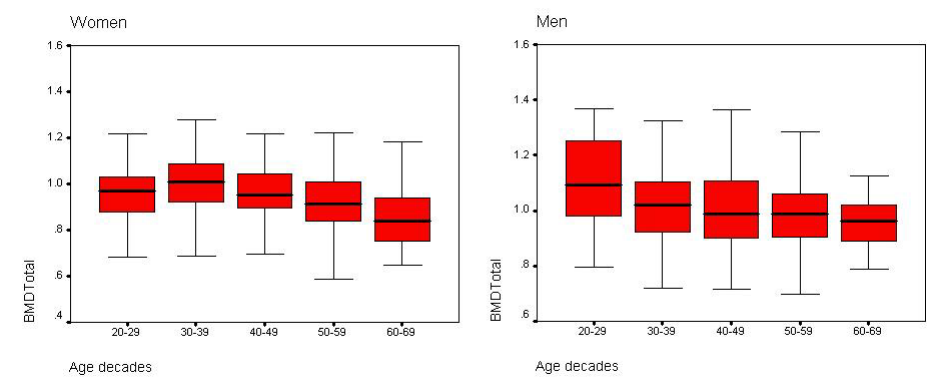
BMD of total hip within age decades and sex (g/cm^2^).

Comparing of BMD between different age decades showed that; spinal BMD in age group of 40–49, 50–59 and 60–69 in women, 3%, 1% and 4% was less than previous age decade respectively. In the same age groups, spinal BMD in men 1%, 6% and 0% was less than previous age decade. Also, femur BMD in age group of 40–49, 50–59 and 60–69 in women, 5%, 5% and 9%, was less than previous age decade, and in men 3%, 4% and 3% was less than previous age decade respectively. (Figure 1, 2).

A significant relationship was found between age and BMD (P < 0.001 and P < 0.001). There was a significant relationship (P = 0.025) between BMD and BMI among the men but there was no relationship of this kind among the women. On the other hand, there was a significant relationship between BMI and femur BMD among both the women and the men (P < 0.001 and P < 0.001 respectively). The loss of BMD among the women was seen more than after menopause so that during the first ten years after the menopause, BMD of the spinal column and femur bone was 1.16 and 2.2 less than each year respectively. In this study, the mean femur BMD among the women with menopause was 10.5 % lower than the women without menopause (P < 0.001). The mean spinal BMD among the women with menopause was 16% lower than the women without menopause (P < 0.001). In total, 7.4% of all cases in lumbar spine and 2.4% in femur bone had osteoporosis and 30.4% in the spinal column and 23.9% in femur bone had osteopenia. (Table 3) show the prevalence of osteoporosis and osteopenia in both sexes and age groups.

**Table 3:** Prevalence of osteoporosis osteopenia in each age and sex groups

			**Osteoporosis**(%)		**Osteopenia**(%)	
			
**Age groups**		**Numbers**	**Spinal column**	**Femur**	**Spinal column**	**Femur**
**20–29**	Women	44	0	2.2	13	17.4
	Men	27	3.8	0	23.1	15.4
**30–39**	Women	104	0	0.9	13.9	8.3
	Men	38	2.7	2.7	27	24.3
**40–49**	Women	98	3.2	2.2	29	15.1
	Men	48	4.3	2.1	31.9	31.9
**50–59**	Women	82	21.8	2.6	46.2	38
	Men	42	5.3	5.3	42.1	36.8
**60–69**	Women	36	32.4	5.9	50	50
	Men	34	9.4	3.1	50	46.9

## Discussion

In this study, the mean of BMD from spinal column and femur in all age groups of men were more than that of women. Most similar studies justify these results through comparing the fluctuations of androgen level with estrogen level, in men the level of androgen to estrogen does not reduce compared with women. On the other hand, the bone mass, physical activities and peak bone mass in men were more than women [25,26]. These results also indicate that the peak bone mass among women were in age of 30–39 which is in accordance with other studies [4-7,25-27]. The mean of spinal column BMD among women 30–39, was 5.6%lower than the American women, 3.9% more than the Japanese women 6.5% more than Filipino women,,0.06% more than Lebanese women [28,29]. There was no significant difference between these two groups because maximum BMD of Japanese occurs in women of 40–49 [26]. Many studies have demonstrated that alteration in BMD depends on type of the bone, different function, menstruation condition, environmental factors, genetics effects and age [30,31]. As the results achieved in other countries indicate different means and amounts, the information obtained through this study show a similar BMD pattern. The present study suggests that the maximum BMD of femur bone compared with spinal column occur later. This is justifiable considering the fact that the maximum BMD in cortical bone compared with trabecular bone occurs later [27,30,31]. The amount of BMD of spinal column was lower of 15.6% after ten years, and this reduction was 16.9% more than the Japanese women [33,35]. The Peak bone mass of femur bone was 4.48% less than the American women [34]. The pattern of bone loss, both in femur and lumbar, depends on the age. The pace of loss in bone mass up to menopause period, in the women in question, is similar to the Canadian, British and American women. Although after that period, it is faster than the women of Belgium, United Kingdom, France and America whereas it is less than the Japanese women [32,33].

On the other hand, the rate of bone loss compared with the other studies (Western Belgian, Japanese,) is either the same or more [32-35]. This trend, therefore, caused an increase in incidence of osteoporosis and osteopenia. In men, the peak bone mass in spinal column was 3.5% less than the American men [7]. The incidence of osteoporosis and osteopenia among women and men was 32.4% and 9.4% respectively, which is more than of Lebanese and Thai women and less than Hong Kong and US women, this is justifiable with regard to the above-mentioned explanations. Studies indicate that the peak bone mass plays an important role the incidence of osteoporosis which this peak bone mass depends on genetics, kind of diet, sport and the hormonal state. Genetics was the most important factor justifying low BMD in our study. As well, with respect to the deficiency of vitamin D in Iran, which is common among 80% of people in some areas, and also lack of enough activity, in particular, among young girls of 20–29 can cause the low level of bone mass [7].

## Conclusion

It is notice-worthy that this paper represents the early results of the comprehensive plan for prevention, diagnosis and treatment of osteoporoses carried out in the EMRC of Tehran University of Medical Sciences, which is still being conducted and is not finished yet. With completing the project and data gathering and studying all patients fully, the final results could be achieved and the relationships could be analytically discussed. Broadly speaking, the present study indicates the high incidence of osteoporosis and osteopenia among the Tehran population, which requires our proper attention and planning for prevention. Also, the low amount of peak bone mass in the ages 20–39 is helpful to adopt an adequate strategy in this respect. There are many factors involved in this maximum BMD including genetic factors, body activity and providing enough vitamin D and calcium.

Among the intervening factors, enough nutrition together with calcium and vitamin D could be enumerated. The results of this study indicate that there is an increase in bone mass in the first decade after menopause requiring a proper treatment during the years before and after menopause.

The limitations of this study are the following points. This is the primary result of the national comprehensive study of osteoporosis in Iran. The individuals excluded from this study based on personal interview and their statement about their disease, not documented diagnostic diseases. In addition, the data of this study limited to Tehran and, our data assumed as an estimation of reference data in Iran. For demonstration of Iran's data, a bigger study is needed.

## List of abbreviations

EMRC: Endocrinology and Metabolism Research Center, TUMS: Tehran University of Medical Sciences, BMD: Bone Mineral Density, BMI: Body Mass Index, DXA: Dual X-ray Absorption

## Competing interests

The author(s) declare that they have no competing interest.

## Authors' contributions

Conception and study design and coordination: BL, AH, Drafting manuscript and Data analysis: AH, AM, Participation in its sequence alignment: MP, AS, MHB, SZM, RD, Review of manuscript and Important intellectual content: BL, AH, AM, All authors read and approved the final manuscript.

## Pre-publication history

The pre-publication history for this paper can be accessed here:


